# Knockdown of Lymphoid Enhancer factor 1 Inhibits Colon Cancer Progression *In Vitro* and *In Vivo*

**DOI:** 10.1371/journal.pone.0076596

**Published:** 2013-10-02

**Authors:** Wen-Juan Wang, Yu Yao, Li-Li Jiang, Ting-Hua Hu, Jie-Qun Ma, Zi-Jun Liao, Jun-Tao Yao, Dong-Fan Li, Shu-Hong Wang, Ke-Jun Nan

**Affiliations:** 1 Department of Oncology, First Affiliated Hospital of Medical College of Xi’an Jiaotong University, Xi’an, China; 2 Affiliated Shaanxi Provincial Cancer Hospital, College of Medicine, Xi’an Jiaotong University, Xi’an, China; 3 Xi’an Central Hospital, Xi’an, China; The University of Hong Kong, China

## Abstract

Expression of lymphoid enhancer factor 1 (LEF1) is frequently altered in different human cancers. This study aimed to assess LEF1 expression in colon cancer tissues and to explore changed phenotypes, gene expressions, and the possible mechanism after knocked down LEF1 expression in colon cancer cell lines. A total of 106 colon cancer and matched paratumorous normal tissues were used to assess LEF1 expression using immunohistochemistry and qRT-PCR. LEF1 lentivirus was used to knockdown LEF1 expression for the assessment of cell viability, cell cycle distribution, apoptosis, and gene expressions. The nude mouse xenograft assay was performed to detect the effects of LEF1 knockdown *in vivo*. The data showed that the levels of LEF1 mRNA and protein were significantly increased in human colon cancer tissues compared to the matched paratumorous normal tissues and were associated with infiltration depth, lymph node and distant metastases, advanced TNM (tumor-node-metastasis) stages, and shorter overall survival. Furthermore, LEF1 knockdown reduced tumor cell viability, invasion capacity, MMP2 and MMP-9 expression, but induced apoptosis. Nude mouse xenograft assay showed that LEF1 knockdown suppressed tumor formation and growth *in vivo*. In addition, the expression of Notch pathway-related proteins RBP-jκ and Hes1 was reduced in LEF1 knockdown cells. Taken together, LEF1 protein was overexpressed in colon cancer tissues and knockdown of LEF1 expression inhibited colon cancer growth *in vitro* and *in vivo*. These data suggest that targeting of LEF1 expression should be further evaluated for colon cancer prevention and therapy.

## Introduction

Colon cancer is one of the most common cancer in both men and women in the world, accounting for the second cause of cancer death in the United States [1,2] and the fourth in China [3]. Thus, colon cancer still remains a major global public health problem, although there is a significantly worldwide decline in cancer-related mortality of colon cancer over the last few decades, due to the considerable progress in early diagnosis and effective treatments. To date, the pathogenesis and molecular mechanism responsible for colon cancer development have been comprehensively studied and a large body of knowledge has been generated regarding molecular alterations associated with colon carcinogenesis, which involves activation of oncogenes and silence of tumor suppressor genes [4]. However, much more needs to be done for the precise understanding of colon carcinogenesis and developing novel strategies to effectively control this disease.

To this end, our research is focusing on lymphoid enhancer-binding factor-1 (LEF1), a member of high mobility group (HMG) protein family. *LEF1* codes a 48-kD nuclear protein that is usually expressed in pre-B and T cells [5,6] and plays an important role in embryogenesis and cancer development [7]. LEF1 is a multifunctional protein that influences numerous cellular functions, such as regulation of Wnt signaling for cell proliferation, apoptosis, mobility, and gene transcription [8,9]. Altered expression of LEF1 protein has been reported to be involved in tumorigenesis of different human cancers, including colon cancer [10]. Molecularly, LEF1 can mediate the expression of Wnt signaling genes via recruitment of β-catenin to the promoter of the target genes [11,12], but LEF1 itself lacks its own transcriptional activation potential in cells. LEF1 protein containing β-catenin binding domains can regulate cell proliferation and invasion of tumor cells [13]. Multiple factors could influence LEF1 expression, such as fibroblast growth factor-2, PITX2, and hepatocyte growth factor [14–16].

Thus, in this study, we first detected LEF1 expression in colon cancer tissues compared with the paratumorous colon tissues and then investigated the effects of LEF1 knockdown in the regulation of colon cancer cell viability, cell cycle distribution, apoptosis, and gene expression *in vitro* and in nude mouse xenografts. We also explored the effects of LEF1 knockdown on the regulation of Notch pathway.

## Materials and Methods

### Ethics Statement

The study was approved by the Conduct of Human Ethics Committee of the First Affiliated Hospital, College of Medicine of Xi’an Jiaotong University. Written informed consents were obtained from all patients.

The animal experimental protocol was approved by the Animal Care and Use Committee of the Medical School of Xi’an Jiaotong University.

### Patients and samples

In this study, we retrospectively recruited 106 pairs of surgically resected colon cancer and paratumorous normal tissue specimens (5 cm away from the tumor lesion) from The First Affiliated Hospital, College of Medicine of Xi’an Jiaotong University between January 2006 and March 2007. These tissue samples were obtained from 60 male and 46 female patients with a mean age of 55.5 years (range from 30 to 81 years). Clinicopathological features of these patients are shown in Table 1. Pathological diagnosis of these specimens was independently re-confirmed by two pathologists in a blinded fashion. All patients were not treated with any chemotherapy or radiotherapy before surgery. The last patient follow-ups were conducted at the end of May 2012. The patients who were lost to follow-up or death from causes other than colon cancer were regarded as censored data during the survival analysis.

**Table 1:** Association of LEF1 expression with clinicopathological factors from patients.

Clinicopathological variables	N	LEF1 expression score (mean ± SD)	*P* value
Age (years)			
<60	41	4.87 ± 2.33	0.502
≥60	65	5.21 ± 2.65	
Tumor differentiation			
Well	50	4.84 ± 1.94	0.274
Moderate	39	4.95 ± 2.87	
Poor	17	5.59 ± 3.02	
Infiltration depth			
T_1_ + T_2_	40	4.12 ± 1.98	0.002
T_3_ + T_4_	66	5.66 ± 2.56	
Lymph node metastasis			
N_0_	46	4.06 ± 2.12	<0.001
N_1-3_	60	5.85 ± 2.74	
Distant metastasis			
M_0_	86	4.69 ± 2.04	0.004
M_1_	20	6.31 ± 2.77	
TNM stage			
I	8	1.39 ± 3.23	<0.001
II	33	4.14 ± 2.40	
III	45	5.33 ± 2.25	
IV	20	7.12 ± 2.69	

### Histopathology and immunohistochemistry

Paraffin-embedded sections (5 µm) were deparaffinized and rehydrated through a series of graded alcohols. For immunohistochemistry, a primary antibody against LEF1 (C12A5, Cell Signaling Technology, Danvers, MA) was used at a dilution of 1:100, and incubated overnight at 4°C. The sections were incubated with an isotype-matched control antibody as a negative control. Next, the sections were stained with a biotin-conjugated secondary antibody and the color was developed by using 0.05% 3’, 3’-diaminobenzidine tetrahydrochloride followed by counterstaining with hematoxylin. The sections were finally reviewed and scored under an Olympus microscope (BX41; Tokyo, Japan), according to a previous study using multiplying the intensity score and the extent of staining score [17]. The staining intensity was scored as 0 (no staining), 1 (weak staining), 2 (medium staining), or 3 (strong staining). The extent of staining was evaluated by assigning samples scores based on the percentage of positively stained cells as follows: 0 (0%), 1 (1-25%), 2 (26-50%), 3 (51-75%), and 4 (76-100%). The number of positive cells was assessed by counting 10 random fields at ×400 magnification. The final score was ranged from 0 to 12. A score of 0-2 was defined as negative expression, scores 3-5 as “weak expression”, scores 6-9 as “moderate expression” and scores 10-12 as “strong expression”. For the purpose of further analysis the samples with score 0-5 were defined as markedly reduced staining or loss of LEF1 expression, while the samples with scores 6-12 were grouped and defined as positive expression.

### Real-time reverse transcription–polymerase chain reaction

Total cellular RNA was isolated from tissues and cell lines using the TRIzol reagent (Invitrogen, Carlsbad, CA), according to the manufacturer’s instructions. These RNA samples were then reversely transcribed into cDNA using an RT-PCR kit (Takara, Dalian, China). Real-time PCR was then performed in iQ5 Multi-color Real-Time PCR Detection System (Bio-Rad, Hercules, CA) using SYBR Premix Ex Taq TM II (Takara). The primer sequences to amplify LEF1 were: 5'-AGCGAATGTCGTTGCTGAGTGTA-3' and 5'-CTCTTGCAGACCAGCCTGGATAA-3'. A housekeeping gene GAPDH was used as the internal control, and the primer sequences were 5'-ACCACAGTCCATGCCATCAC-3' and 5'-TCCACCACCCTGTTGCTGTA-3'. The data were acquired as a threshold cycle (ΔC_t_) value. The ΔC_t_ values were determined by subtracting the average internal housekeeping gene C_t_ value from the average target gene C_t_ value. Since the amplification efficiency of the target genes and internal control gene was equal, the relative gene expression was calculated using the 2^-ΔΔCt^ method. Each measurement was performed in triplicate.

### Construction of lentivirus vector carrying LEF1 shRNA

A LEF1 shRNA lentivirus vector (U6-vshRNA-CMV-PUR-GFP-GV248-shLEF1) was designed, synthesized, and constructed by Genechem Co. (Shanghai, China). The following target sequences in the human LEF1 gene were selected, i.e., target 1, GCTGACATCAAGTCTTCCTTG; target 2, GTGAAGAGCAGGCTAAATATT; target 3, GCTGGTCTGCAAGAGACAATT; and target 4, GCTCA TTCCCAACGTGCAAAG. Target 3 was chosen for the subsequent experiments. The lentivirus vector U6-vshRNA-shNC, which encodes a random RNAi sequence, was used as a negative control.

### Cell lines, culture, gene transfection and treatments

Human colon cancer cell lines, Caco_2_, colo205, HCT116, HT29, and Lovo were obtained from Cell Resource Center, Shanghai Institutes for Biological Sciences (Shanghai, China) and cultured in Dulbecco’s modified Eagle medium (DMEM) (HyClone, Logan, UT, USA) supplemented with 10% fetal bovine serum (FBS; Invitrogen), penicillin (100 IU/ml), and streptomycin (0.1 mg/ml) in 5% CO_2_ at 37°C. A human colon cancer cell line SW480 was cultured in RPMI1640 medium (HyClone, Logan, UT, USA) supplemented with 10% FBS, penicillin (100 IU/ml), and streptomycin (0.1 mg/ml) in 5% CO_2_ at 37°C, while another human colon cancer cell line, SW620, was cultured in L-15 medium (Invitrogen) supplemented with 10% FBS, penicillin (100 IU/ml), and streptomycin (0.1 mg/ml) in 5% CO_2_ at 37°C. SW480 and SW620 cells were infected with lentivirus vectors at a multiplicity of infection (MOI) of 10 and 100, respectively, and then selected in puromycin-containing growth medium. After 7 days of culture, puromycin-resistant colonies were picked up, expanded and analyzed separately.

γ-secretase inhibitor DAPT (100 µM; Sigma-Aldrich, USA) was used for pharmacological inhibition assays, which can effectively block Notch intracellular domain (NICD) entering into the cell nucleus. In brief, these cells were seeded into six-well plates and treated with 2 ml medium containing 1% FBS and DAPT. Control cells were treated with equal amounts of dimethyl sulfoxide (DMSO). After 48h of incubation, the cell extracts were prepared for Western blot as described below.

### Protein extraction and Western blot

Protein was extracted from the tissues and cells, and these protein lysates were separated by 6%-12% sodium dodecylsulfate polyacrylamide gel electrophoresis; separated proteins were then electronically blotted onto polyvinylidene difluoride membranes (Millipore, Danvers, MA). The membranes were then blocked and subsequently incubated with the following primary antibodies: anti-LEF1 antibody (1:800; Cell Signaling Technology, Danvers, MA), anti-MMP2 (1:500; Cell Signalling Technology, Danvers, MA), anti-MMP7 (1:100; Santa Cruz Biotechnology, Santa Cruz, CA), anti-MMP9 (1:100; Santa Cruz Biotechnology, Santa Cruz, CA), anti-NICD (1:800; Cell Signalling Technology), anti-RBP-jκ (1:100; Santa Cruz Biotechnology, Santa Cruz, CA), anti-Hes1 (1:50; Santa Cruz Biotechnology), or anti-β-actin antibody (1:1000; Santa Cruz Biotechnology). At last, the blots were visualized by an ECL detection system (Millipore) with a horseradish peroxidase-conjugated secondary antibody (Santa Cruz Biotechnology). Western blots were repeated three times for each protein sample.

### Cell viability MTT assay

Cells (5×10^3^ per well) were seeded with 200 µl of growth medium in a 96-well plate and grown up to 7 days. At the end of the experiments, 3-[4,5-dimethylthiazol-2-yl]-2,5-diphenyl-tetrazolium bromide (MTT, 0.5mg/ml, Sigma-Aldrich, St. Louis, MO, USA) was added to each well. The cells were then cultured at 37°C for 4h, and 150 µl DMSO was added into each well and mixed by shaking at room temperature for 10 min. After that, the absorption rate was measured at a wavelength of 490 nm using a spectrophotometer. Each experiment was done in triplicate and repeated at least three times.

### Flow cytometry cell cycle and apoptosis assays

For cell cycle analysis, cells (5×10^5^) were grown and collected, washed two times with phosphate buffered saline (PBS), and fixed with ice-cold 70% ethanol for 24 h at 4°C. The fixed cells were stained with 150 µl propidium iodide and 150µl RNase A (Sigma-Aldrich) sequentially. After incubation for 30 min at 37°C, the samples were examined by a flow cytometer (FACS-Caliber, Franklin Lakes, NJ), and Cell Quest software was used to analyze the data. Each experiment was repeated at least three times.

For apoptosis analysis, cells (5×10^5^ per well) were cultured in six-well plates, collected, and washed two times with ice cold PBS. Next, 500 µl of the binding buffer, 5 µl Annexin V-APC, and 5 µl 7-AAD (KeyGEN BioTECH, Nanjing, China) were sequentially added to the samples. After mixing and incubation for 15 min at 37°C, the samples were run on the flow cytometry immediately. Each experiment was repeated at least three times.

### Tumor cell Matrigel invasion assay

The invasion capacity of colon cancer cells was assessed by using a Millicell invasion chamber (Millipore, Billerica, MA, USA). The 8 µm pore inserts were coated with Matrigel (Becton and Dickinson Company, Franklin Lakes, NJ, USA). Cells (5×10^4^) were re-suspended with 200 µl of serum-free medium with 5 µg/ml mitomycin C (Sigma-Aldrich) into the top chamber, and the bottom chamber was filled with normal culture medium. After incubation for 24 h, the non-invading cells on the upper surface were removed with a cotton-tipped swab and the invading cells on the bottom surface of filter were fixed in methanol, stained with 0.1% crystal violet, and counted under a microscope using 10 randomly selected fields in a magnification of 200x.

### Nude mouse tumor cell xenograft assay

Male BALB/c nude mice (ages between 4 and 6 weeks; Shanghai Experimental Animal Center, Shanghai, China) were subcutaneously inoculated with 1×10^7^ cells and housed in a pathogen-free facility of the Animal Center of Xi’an Jiaotong University. The tumor volume was determined by length (L) and width (W), measured with a sliding calliper and calculated as L × W^2^×0.5. The mice were euthanized 45 days after the subcutaneous injection of tumor cells. Tumor bearing nude mice were observed by IVIS imaging system (IVIS spectrum, Xenogen, CA, USA) before being sacrificed.

### Statistical analysis

All statistical analyses were performed using the SPSS 17.0 software (SPSS Inc., Chicago, IL). Descriptive data were analyzed with Pearson’s chi-square test (two sided). Measurement data were analyzed with Student’s *t*-test or analysis of variance (ANOVA). A *P* value of less than 0.05 was considered statistically significant.

## Results

### LEF1 expression in human colon cancer tissues and cell lines

**Figure 1:**
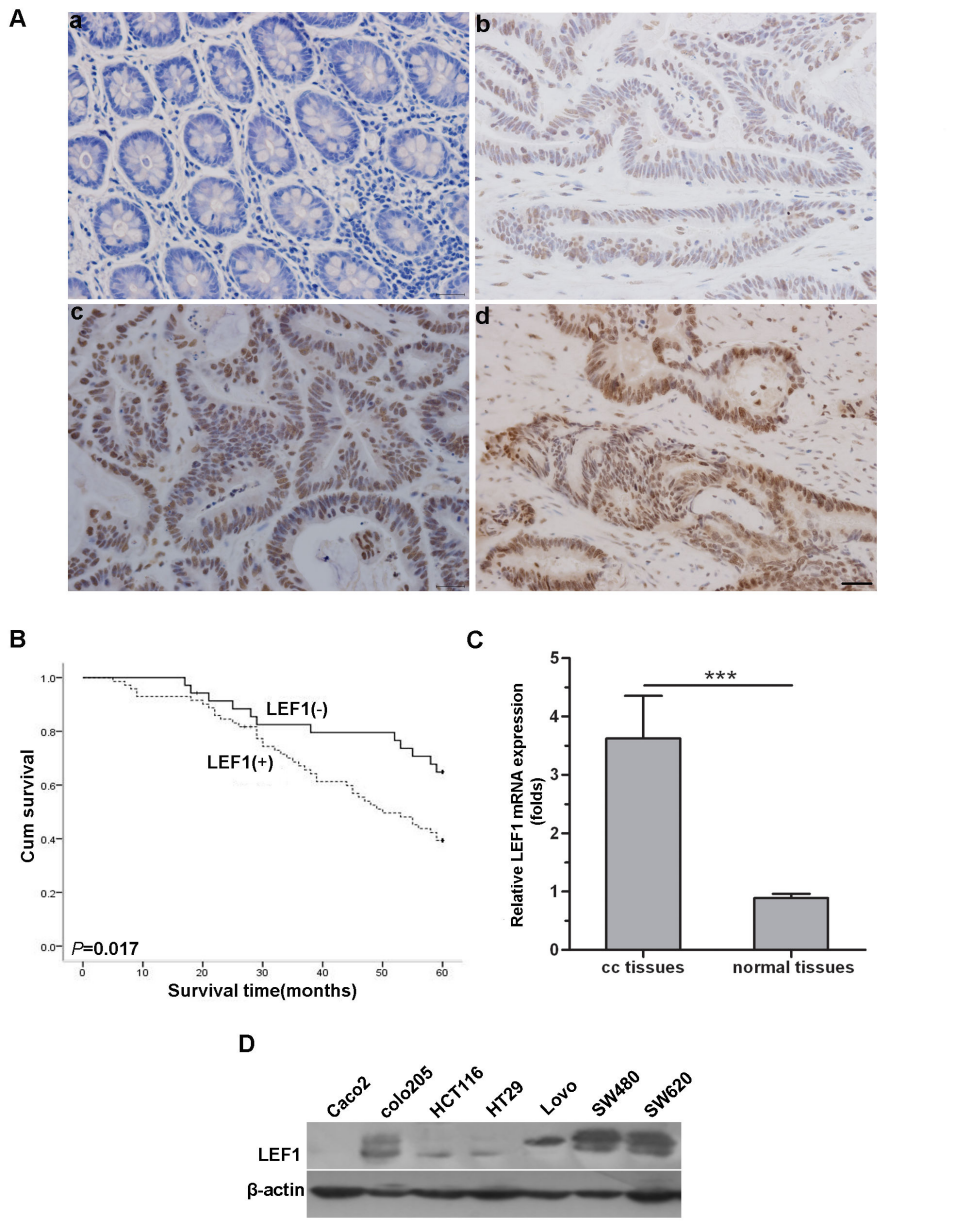
Expression of LEF1 mRNA and protein in colon cancer tissues, paratumorous colon tissues and colon cancer cell lines. (A) Immunohistochemistry staining of LEF1 on paratumorous colon tissues (a) and colon cancer tissues (b-d) (scale bar, 25µm): (a) negative expression, (b) weak expression, (c) moderate expression, (d) strong expression. (B) Kaplan-Meier curve for association of LEF1 protein expression with overall survival of patients. (C) qRT-PCR was used to detect the relative expression levels of LEF1 mRNA (cc tissues: colon cancer tissues; normal tissues: paratumorous colon tissues). Columns, mean (n=106); bars, SD; ***P<0.001 compared with paratumorous colon tissues. *P* value was determined by Student’s *t*-test. (D) Western blot analysis of LEF1 expression in seven colorectal cancer cells (Caco_2_, colo205, HCT116, HT29, Lovo, SW480, and SW620). β-actin was used as internal control.

In this study, we first determined expression of LEF1 protein in human colon cancer tissues and cell lines using immunohistochemistry. The results showed that 71 of 106 colon tissues and 23 of 106 paratumours normal colon tissues expressed the LEF1 protein, indicating that colon cancer tissues expressed higher levels of LEF1 than those in the paratumours normal colon tissues (*P*<0.05; Figure 1A). Obviously, LEF1 expression was observed in the nuclei in colon cancer tissues and paratumours normal colon tissues (Figure 1A). Moreover, expression of LEF1 protein was associated with infiltration depth, lymph node and distant metastases, and advanced TNM (tumor-node-metastasis) stages of colon cancer (*P*<0.01; Table 1). The survival analysis (5-year follow-up of 106 colon cancer patients) showed that the median survival rate of patients with LEF1 expressed tumor was 48.5 months, which was significantly poorer than those with LEF1-negative tumors (more than 60 months) (*P*<0.05; Figure 1B).

Similarly, real-time PCR analysis showed that levels of LEF1 mRNA were significantly increased in these 106 pairs of colon cancer tissues compared to the paratumours normal colon tissues (*P*<0.05; Figure 1C). Furthermore, LEF1 expression was highly expressed in colon cancer cell lines SW480 and SW620 (Figure 1D). Thus, we chose these two cell lines to knockdown LEF1 expression for further study.

### Stable knockdown of LEF1 expression in colon cancer cells

**Figure 2:**
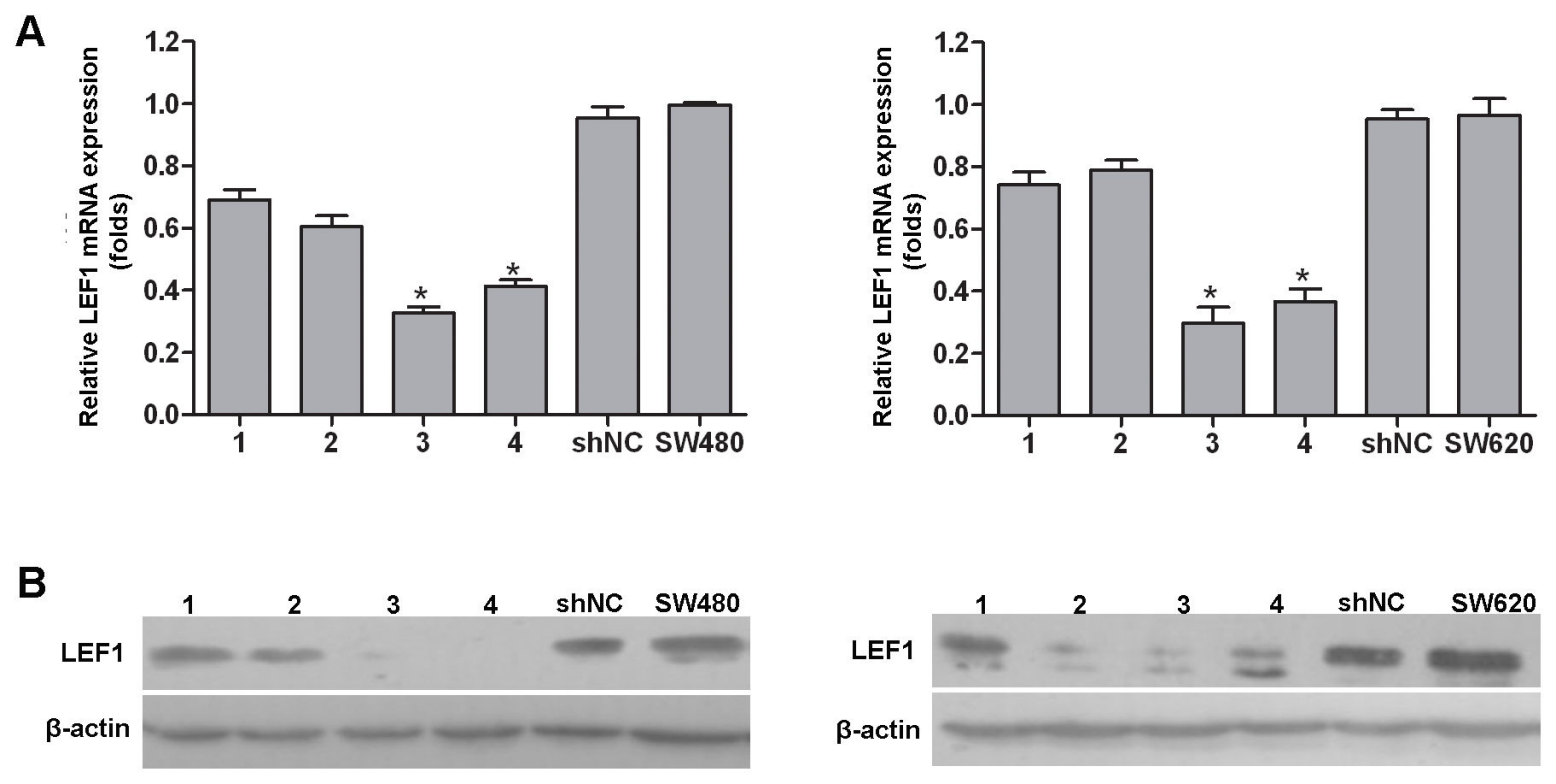
Establishment of stable LEF1 knockdown cell lines. (A) qRT-PCR analysis of LEF1 expression after infection of different LEF1 shRNA expression lentivirus vectors, *P<0.05 compared to the parent cell. (B) Western blot analysis of LEF1 protein expression. β-actin was used as internal control. 1-4 (different target position vectors) and shNC (negative control). Target 3 was used to establish the stable LEF1 knockdown cell lines.

To explore the role of LEF1 on colon cancer cells, we utilized a lentivirus carrying LEF1 shRNA to infect SW480 and SW620 for knockdown of LEF1 expression. Target 3 was used to establish the stable LEF1 knockdown cell lines. We successfully obtained stable LEF1 knockdown cells termed shLEF1 and shNC (with negative control vector). Real-time PCR analysis showed that the expression of LEF1 mRNA was inhibited up to 70% in shLEF1 cells compared to shNC cells (*P*<0.05; Figure 2A). Similarly, the levels of LEF1 protein were also knocked down significantly in shLEF1 cells than in shNC cells (Figure 2B).

### Knockdown of LEF1 expression inhibited viability of colon cancer cells *in vitro* and tumor formation and growth *in vivo*

**Figure 3:**
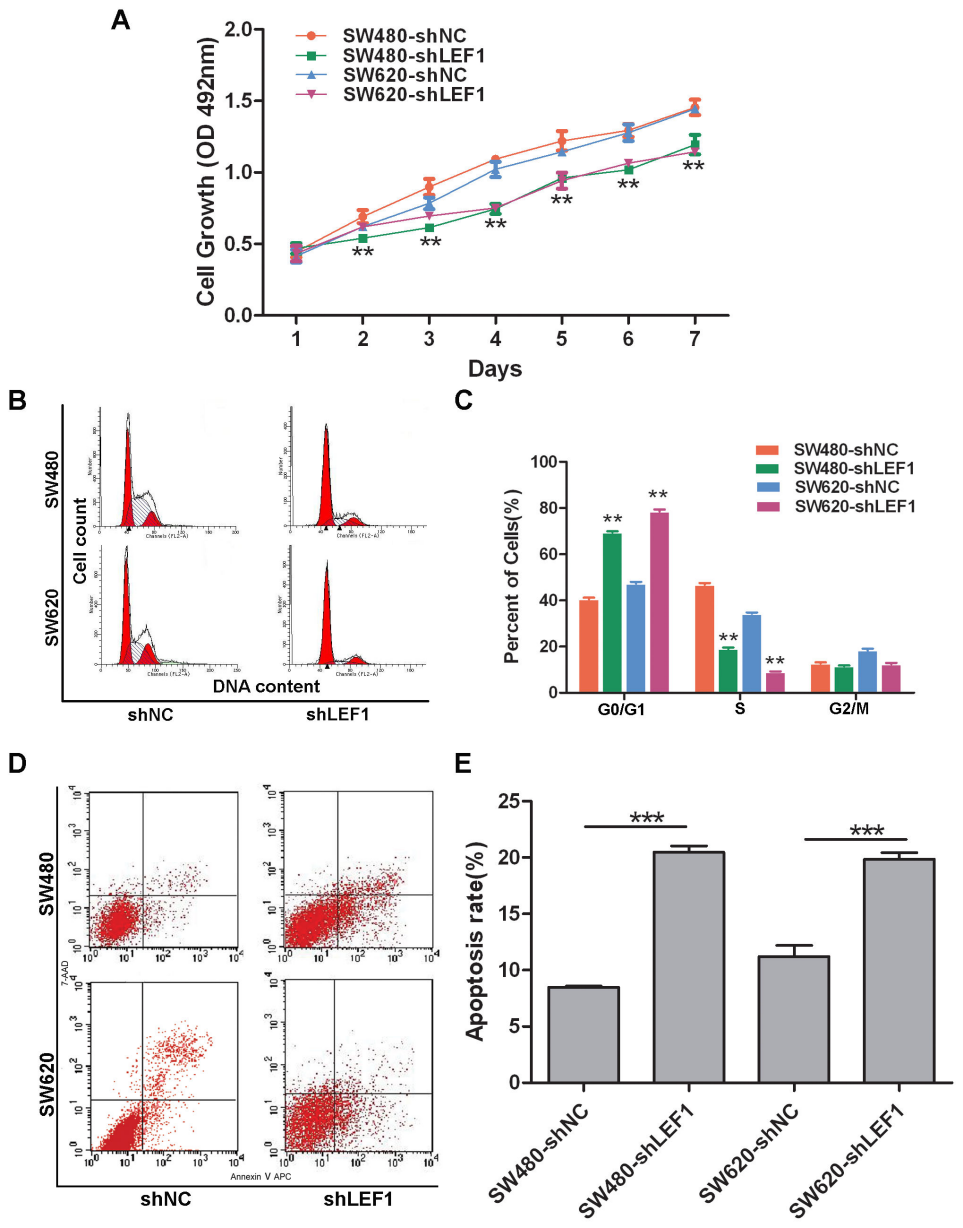
Effects of LEF1 knockdown on inhibition of colon cancer cell viability *in vitro*. (A) MTT assay. **P<0.01 compared with the corresponding shNC cells. (B and C) Flow cytometric cell cycle distribution. Columns, mean (n=3); bars, SD; **P<0.01 compared with the corresponding shNC cells. (D and E) Flow cytometric apoptosis assay. Cell spontaneous apoptosis was assessed by flow cytometry. Columns, mean (n=3); bars, SD; ***P<0.001 compared to the control shNC cells.

Stable LEF1 shRNA transfectants were seeded and grown in the puromycin-containing growth medium for the assessment of cell viability. Our data showed that SW480-shLEF1 cells and SW620-shLEF1 cells grew much slower than SW480-shNC cells and SW620-shNC cells, respectively (*P*<0.01; Figure 3A). Cell cycle distribution detected by a flow cytometer showed a prolonged and prominent delay of G0 / G1 phase progression to S phase in shLEF1 cells compared to that of shNC cells (*P*<0.01; Figure 3B and 3C). Moreover, we further analyzed whether the reduced tumor cell viability is due to induction of apoptosis and found that SW480 and SW620 cells with stable LEF1 shRNA transfection had significantly more apoptosis compared to the control cells (Figure 3D and 3E).

**Figure 4:**
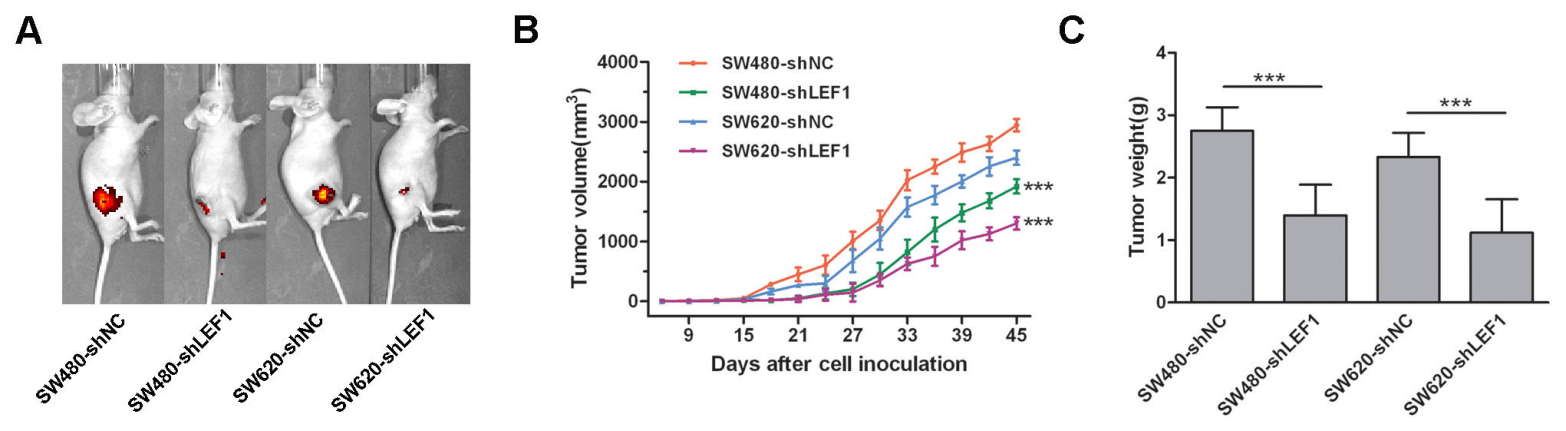
Effects of LEF1 knockdown on regulation of formation and growth of nude mouse xenografts. (A) *In* *vivo* imaging analysis. Growth of tumors formed by shLEF1 cells and control shNC cells in nude mice was imaged by IVIS. (B) Tumor volume was measured every 3 days from day 9 after the inoculation by measuring tumor length and width. Columns, mean (n=6); bars, SD; ***P<0.001 compared to the control shNC cells. (C) Tumor weight was compared on day 45 after tumor cell inoculation. Columns, mean (n=6); bars, SD; ***P<0.001 compared to the control shNC.

Furthermore, we performed a nude mouse xenograft assay to assess the role of LEF1 knockdown *in vivo*. Forty five days after tumor cell injection, LEF1 knockdown-tumor xenografts showed a reduction of tumor growth compared to control-shNC tumor xenografts. Additionally, *in vivo* imaging showed that both types of mice did not have observable tumor metastases to the distant organs (Figure 4A). The average volumes of the tumor mass derived from SW480-shLEF1 cells and SW620-shLEF1 cells were much smaller than those of tumor xenografts derived from SW480-shNC cells and SW620-shNC cells, respectively (*P*<0.001; Figure 4B). The tumor weight also showed that knockdown of LEF1 expression inhibited the growth of colon cancer cells in nude mice (Figure 4C), because the weight of tumors mass in SW480 and SW620 cells expressing shLEF1 was significantly lighter than those in the control mice. These finding suggested that LEF1 knockdown inhibited formation and growth of tumor xenografts in vivo.

### Knockdown of LEF1 expression decreased invasion of colon cancer cells and expression of MMP-2 and MMP-9

**Figure 5:**
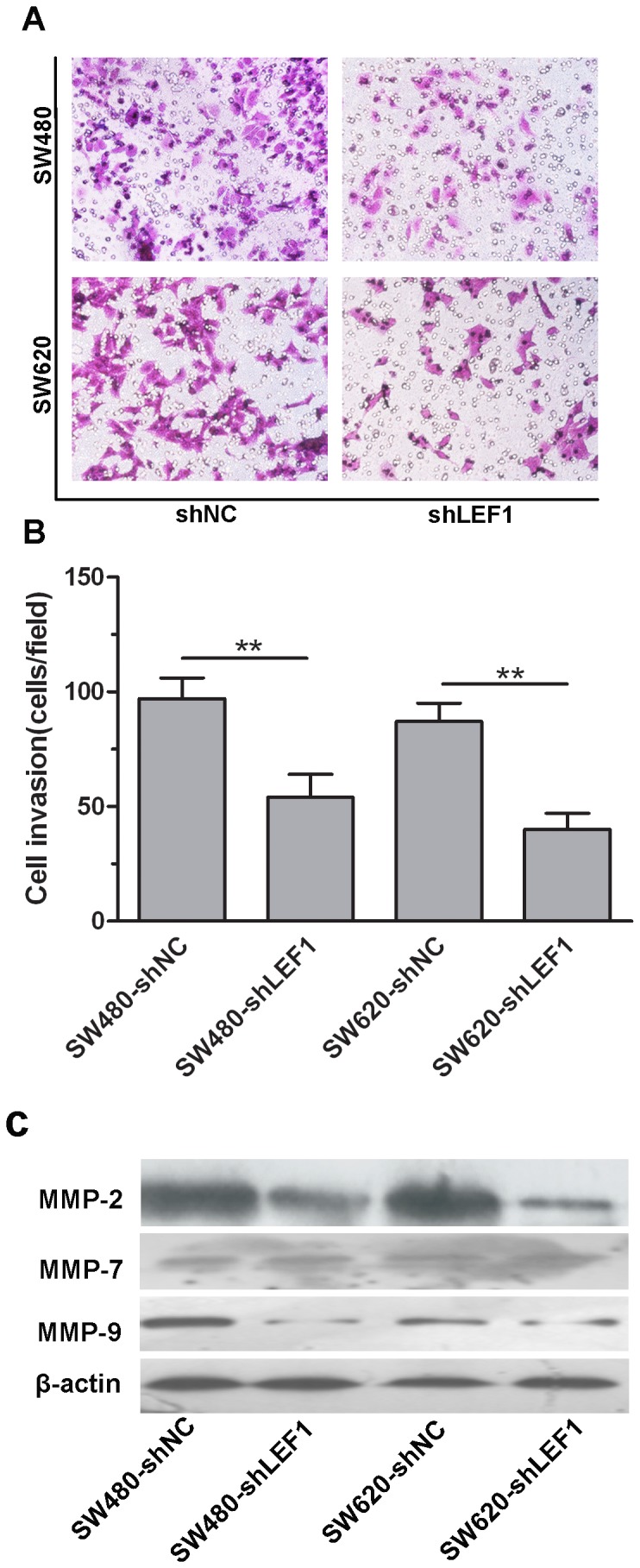
Effects of LEF1 knockdown on regulation of tumor cell invasion and expression of MMP2 and MMP9 proteins *in vitro*. (A and B) Tumor cell invasion assay. Representative staining images, ×200. Quantitative analysis of invaded tumor cells between LEF1 knockdown and control cells. Columns, mean (n=3); bars, SD; **P<0.01 compared to the control shNC cells. (C) Western blot analysis to MMP2, MMP-7 and MMP-9 protein. β-actin was used as internal control.

After that, we determined the effect of LEF1 knockdown on the regulation of colon cancer cell invasion using the Matrigel invasion assay. We treated the cells with Mitomycin C to eliminate the influence of increased proliferation. The results showed that the numbers of invading SW480-shLEF1 cells and SW620-shLEF1 cells were much lower than that of SW480-shNC cells and SW620-shNC cells (*P*<0.01; Figure 5A and 5B). Moreover, knockdown of LEF1 expression could significantly decrease the levels of MMP-2 and MMP-9 protein, but not MMP-7 protein in shLEF1 cells compared to that of shNC colon cancer cells (Figure 5C).

### Knockdown of LEF1 expression inhibited RBP-jκ activity

**Figure 6:**
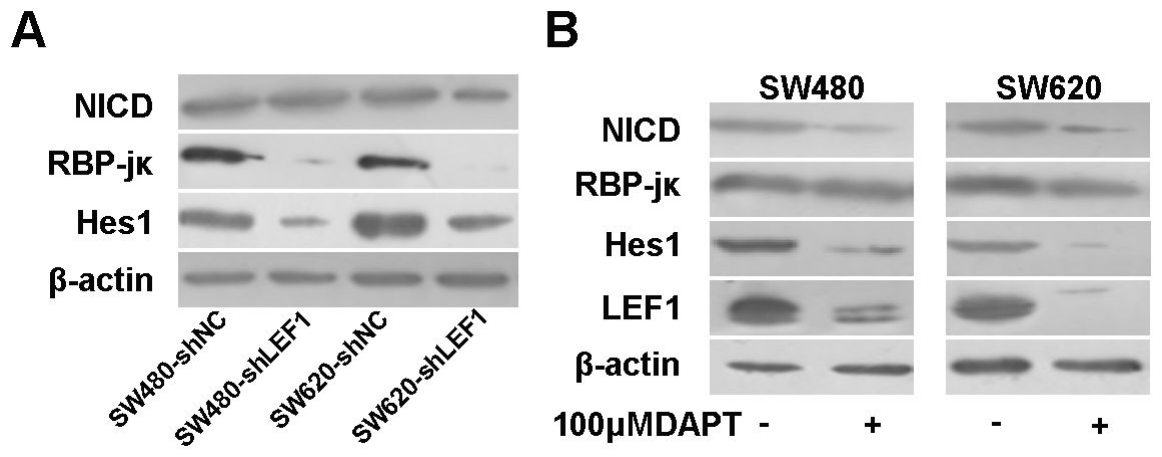
Effects of LEF1 knockdown on regulation of Notch pathway gene expression. (A) Western blot analysis of NICD, RBP-jκ and Hes1 expression. (B) Western blot analysis of LEF1 expression after being treated with Notch pathway inhibitor DAPT (100 µM). β-actin was used as internal control.

To date, several studies have reported that Wnt and Notch pathway cooperatively control cell proliferation and tumorigenesis in the intestines [18]. To disclose the possible convergent points and to clarify the potential mechanism by which LEF1 regulates proliferation and tumorigenesis, we detected the expression of Notch intracellular domain (NICD)/RBP-jκ/Hes1 pathway genes in these colon cancer cells. We did not find any significant difference in levels of NICD expression in colon cancer cells with different treatments; however, the levels of RBP-jκ and Hes1 proteins were reduced in SW480-shLEF1 cells and SW620-shLEF1 cells compared with control cells (Figure 6A).

Our unpublished data showed an increased activity of full-length LEF1 promoter upon co-transfection of NICD cDNA in SW480, HepG2, HEK293, A549, and HeLa cells; thus, we utilized DAPT, an inhibitor of Notch pathway, to block NICD expression in SW480 and SW620 cells. Our data showed that NICD downregulation affected expression of Hes1 and LEF1 proteins (Figure 6B), suggesting a reciprocal regulation between LEF1 and Notch pathway.

## Discussion

In the current study, we first analysed the expression of LEF1 mRNA and protein in colon cancer tissue specimens and then investigated the effects of LEF1 knockdown on the regulation of changed tumor cell viability, cell cycle distribution, apoptosis, and gene expressions *in vitro* and on nude mouse xenografts. We found that levels of LEF1 mRNA and protein were significantly increased in colon cancer tissues and associated with infiltration depth, lymph node and distant metastases and shorter overall survival. LEF1 knockdown reduced tumor cell viability, invasion capacity, and expression of MMP2 and MMP-9, but induced apoptosis in colon cancer cells. LEF1 knockdown suppressed tumor formation and growth in nude mice. Moreover, the expression of RBP-jκ and Hes1 was reduced in LEF1 knockdown cells. These data suggest that LEF1 could be further evaluated as a target for colon cancer prevention and treatment.

Previous studies demonstrated that the expression and activation of LEF1 protein contributed to cancer development [10,13,19]. Indeed, our current study analysed the expression of LEF1 mRNA and protein in colon cancer and paratumorous colon tissues and found that LEF1 was overexpressed in colon cancer tissues. The levels of LEF1 expression were associated with infiltration depth, lymph node, and distant metastases, and advanced TNM stages of colon cancers as well as poor overall survival rate in patients with colon cancer. The latter data were different from the data reported by Kriegl, L et al [20], but were similar to another previous study [21]. These data suggest that LEF1 may be a useful marker for the prediction of colon cancer progression.

To further understand the role of LEF-1 in colon cancer, we knocked down LEF1 expression in two colon cancer cell lines, SW480 and SW620. We further explored the role of LEF1 in the growth of colon cancer. Cell cycle disorganisation was defined as an inducer that lead to uncontrolled cell proliferation and cancer progression followed by tumorigenesis, for example, Shtutman et al identified that cyclin D1 was one of the β-catenin/LEF1 complex target genes [22], which was responsible for tumor cell proliferation and tumor progression. Consistent with this study, our current data showed that downregulation of LEF1 significantly inhibited colon cancer cell proliferation *in vitro* by increasing the sub-G1 apoptotic population, prolonging the G0/G1 phase, and reducing G2/S phase in LEF1-knocked down SW480 and SW620 cells *in vitro* and in nude mouse xenografts, which confirmed two other previous studies in human endometrial and colon cancers [9,23].

Furthermore, our current study showed that LEF1 overexpression in the primary CRC tissues was associated with distant metastasis of CRC patients, which is consistent with several previous studies documenting that LEF1 was characterized as a biomarker for colon cancer to metastasize to the liver [21]. Indeed, our current *in vitro* data confirmed that knockdown of LEF1 expression inhibited invasion ability of SW480 and SW620 cells compared with the control shRNA-transfected cells. At the molecular level, MMP2, MMP7 and MMP9 are associated with cell migration process, influencing cancer development and progression [24,25]. We found that knockdown of LEF1 expression suppressed MMP2 and MMP-9 expression, but not MMP-7, indicating that the selective modulation of MMPs by LEF1 could have the biological significance in colon cancer progression. In contrast, LEF1 was able to regulate MMP-7 expression and activity in breast cancer cells [26], whereas another previous study showed that circulating MMP-2 and MMP-9 could be used to potentially classify patients into low risk, high risk, benign disease, and breast cancer [24]. However, our nude mouse xenograft assay did not show any tumor metastasis in both the LEF-1 knockdown and the control tumor xenografts; thus, additional studies are clearly needed.

In addition, the Notch pathway is frequently activated in various human cancers, such as cancers of the brain, mammary glands, cervix, lung, head and neck, colon, kidney, pancreas, and acute myeloid [27,28]. Inhibition of Notch pathway induced tumor cells to apoptosis and reduced tumor cell proliferation in colorectal cancer [29]. A recent publication showed that the Wnt pathway was able to regulate the Notch pathway [30]. The cross talk between Notch and Wnt pathways may be partially mediated by the specific regulation of GSK-3β-dependent Notch phosphorylation. Phosphorylation of Notch proteins has been indirectly correlated with Notch activation and nuclear translocation as well as cellular transformation. In addition, Jagged1, one of Notch ligands, can be activated by β-catenin during ectopic hair follicle formation in adult epidermis [31]. In our current study, we found that expression of Notch pathway-related genes RBP-jκ and Hes1 were markedly reduced, and the expression level of NICD was not changed after silence of LEF1 protein expression, which may in turn influence the Notch pathway and the Notch target gene, Hes1 expression, and activation. However, because the Notch pathway can be regulated by multiple factors, only blockage of LEF1 expression may not totally inhibit activity of the Notch pathway. Nevertheless, to date, there is no study showing that knockdown of LEF1 expression could inhibit RBP-jκ activity. Indeed, in search of literature, we found that Ungerback et al identified the promoter of RBP-jκ gene indeed contains five putative LEF1/TCF-site [32], which suggested LEF1 may directly regulate expression of RBP-jκ. However, further studies are needed to confirm that they are functional. Furthermore, we also observed that inactivation of Notch by using DAPT reduced LEF1 expression, suggesting that there is an interesting reciprocal regulation of LEF1 and Notch pathway. This finding is consistent with a previous study [33].

However, our current study is just proof-of-principle and much more needs to be done to clarify the function and role of LEF1 in colon cancer development and progression. Further studies will verify LEF1 as a biomarker for the prediction of colon cancer progression and survival of patients. Thereafter, we will evaluate LEF1 as a promising therapeutic target for the prevention and therapy of colon cancer.
